# Supplementary material for: Factors Contributing to Chronic Kidney Disease following COVID-19 Diagnosis in Pre-Vaccinated Hospitalized Patients
Source: Vaccines (Basel). 2023 Feb 13;11(2):433. doi: 10.3390/vaccines11020433 (PMC9966430; doi:10.3390/vaccines11020433)
Supplement: Supplementary file 1 [file vaccines-11-00433-s001.zip › vaccines-2142901-supplementary.pdf]

Table S1: Prevalence and Percentage Antiviral and Antibiotic Used.

No	Antiviral	n (%)	Antibiotic	n (%)
1	Isoprinosinee (3 x 500 mg)	5 (1.3)	Levofloxacin (1 x 750 mg)	66 (17.5)
2	Favipiravir (day 1: 2 x1600 mg days 2-4: 2 x 600 mg)	74 (19.6)	Meropenem (3 x 1 g)	3 (0.8)
3	Remdesivir (1 x 200 mg)	31 (8.2)	Ceftriaxone (1 x 2 g)	13 (3.4)
4	Isoprinosine (3 x 500 mg) + remdesivir (1 x 200 mg)	25 (6.6)	Azitromycin(1 x 500 mg)	43 (11.4)
5	Isoprinosine (3 x 500 mg) + favipiravir (day 1: 2 x1600 mg; days 2-4: 2 x 600 mg) inver	29 (7.7)	Levofloxacin (1 x 750 mg) + Meropenem (3 x 1 g)	17 (4.5)
6	Isoprinosine (3 x 500 mg) + remdesivir (1 x 200 mg) + favipiravir (day 1: 2 x 1600 mg; days 2-4: 2 x 600 mg)	21 (5.6)	Levofloxacin (1 x 750 mg) + Ceftriaxone (1 x 2 g)	37 (9.8)
7	Isoprinosine (3 x 500 mg) + favipiravir (day 1: 2 x 1600 mg; days 2-4: 2 x 600 mg) + remdesivir (1 x 200 mg) + oseltamivir (2 x 75 mg)	3 (0.8)	Meropenem (3 x 1 g) + Azitomycin (1 x 500 mg)	2 (0.5)
8	Favipiravir (day 1: 2 x 1600 mg; days 2-4 2 x 600 mg) + remdesivir (1 x 200 mg)	33 (8.7)	Azitromycin (1 x 500 mg) + Ceftriaxone (1 x 2 g)	11 (2.9)
9	Isoprinosine (3 x 500 mg) + remdesivir (1 x 200 mg) + favipiravir (day 1: 2 x1600 mg; days 2-4: 2 x 600 mg)	1 (0.3)	Fosfomycin (2 x 1 g) + Ceftazdim	1 (0.3)
10	Isoprinosine (3 x 500 mg) + oseltamivir (2 x 75 mg) + Invermectin (1 x 24 mg)	1 (0.3)	Cotrimoxazole (1 x 960 mg) + Ceftriaxone (1 x 2 g)	2 (0.5)

11	No antiviral agent	57 (15.1)	Azitromycin (1 x 500 mg) + Meropenem (3 x 1 g) + Ceftriaxone (1 x 2 g)	2 (0.5)
12	Isoprinosine (3 x 500 mg)+ Remdesivir (1 x 200 mg) + Methisoprinol (3 x 500 mg)	1 (0.3)	Levofloxacin (1 x 750 mg) + Ceftriaxone (1 x 2 g) + Meropenem (3 x 1 g)	5 (1.3)
13	Oseltamivir (2 x 75 mg) + Favipiravir (day 1: 2 x1600 mg; days 2-4 2 x 600 mg) + Remdesivir (1 x 200 mg)	18 (4.8)	Azitromycin (1 x 500 mg) + Meropenem (3 x 1 g) + Levofloxacin (1 x 750 mg)	14 (3.7)
14	Isoprinosine (3 x 500 mg) + Favipiravir (day 1: 2 x1600 mg; days 2-4: 2 x 600 mg) + Oseltamivir (2 x 75 mg)	2 (0.5)	Azitromycin (1 x 500 mg) + Cefotaxime (3 x 1 g) + Meropenem (3 x 1 g)	2 (0.5)
15	Ivermectin + Remdesivir (1 x 200 mg)	1 (0.3)	Levofloxacin (1 x 750 mg) + Ceftriaxone + Meropenem (3 x 1 g) + Amikacin (2 x 500 mg)	1 (0.3)
16	Isoprinosine (3 x 500 mg) + Remdesivir (1 x 200 mg) + Oseltamivir (2 x 75 mg)	3 (0.8)	Fosfomycin (2 x 1 g)+ Doripenem (3 x 1 g)+ Cefixime (2 x 200 mg) + Vancomycin (1 x 1.5 g) + Levofloxacin (1 x 750 mg)	1 (0.3)
17	Isoprinosine (3 x 500 mg) + Remdesivir (1 x 200 mg) + Favipiravir (day 1: 2 x1600 mg; days 2-4: 2 x 600 mg) + Invermectin (1 x 24 mg)	2 (0.5)	Azithromycin (1 x 500 mg) + Levofloxacin (1 x 750 mg) + Ceftriaxone (1 x 2 g)	13 (3.4)
18	Methisoprinol (3 x 500 mg) + Favipiravir (day 1: 2 x 1600 mg; days 2-4: 2 x 600 mg)	1 (0.3)	Azithromycin (1 x 500 mg) + Ceftriaxone (1 x 2 g) + Levofloxacin (1 x 750 mg) + Cefixime (2 x 200 mg)	1 ((0.3)
19	Favipiravir (day 1: 2 x1600 mg; days 2-4 2 x 600 mg) + Oseltamivir (2 x 75 mg)	13 (3.4)	Ceftriaxone (1 x 2 g) + Cefixime (2 x 200 mg)	1 (0.3)

20	Oseltamivir (2 x 75 mg)	7 (1.9)	Ceftazidime (3 x 1 g) + Cotrimoxazole (1 x 960 mg) + Amikacin (2 x 500 mg) + Levofloxacin (1 x 750 mg) + Azithromycin (1 x 500 mg) + Ceftriaxone (1 x 2 g) + Meropenem (3 x 1 g)	1 (0.3)
21	Favipiravir (day 1: 2 x 1600 mg; days 2-4 : 2 x 600 mg) + Oseltamivir (2 x 75 mg) + Isoprinosine (3 x 500 mg)	8 (2.1)	Azithromycin (1 x 500 mg) + Cefotaxime (3 x 1 g)	3 (0.8)
22	Favipiravir (day 1: 2 x 1600 mg; days 2-4 2 x 600 mg) + Oseltamivir (2 x 75 mg) + Isoprinosine (3 x 500 mg) + Remdesivir (1 x 200 mg)	32 (8.5)	Azithromycin (1 x 500 mg) + Levofloxacin (1 x 750 mg)	27 (7.1)
23	Oseltamivir (2 x 75 mg) + Isoprinosine (3 x 500 mg)	9 (2.4)	Cefotaxime (3 x 1 g)	2 (0.5)
24	Oseltamivir (2 x 75 mg) + Remdesivir (1 x 200 mg)	1 (0.3)	Lefofloxacin (1 x 750 mg) + Ceftriaxone + Azithromycin (1 x 500 mg) + Meropenem (3 x 1 g)	4 (1.1)
25	Total	378	Levofloxacin (1 x 750 mg) + Ceftazidime (3 x 1 g) + Cefotaxime (3 x 1 g)	1 (0.3)
26			Clindamycin (3 x 300 mg)+ Levofloxacin (1 x 750 mg)	1 (0.3)
27			Cefotaxime (3 x 1 g)+ Cefixime (2 x 200 mg)	1 (0.3)
28			No Antibiotic	29 (7.7)
29			Azithromycin (1 x 500 mg) + Cefoperazone (2 x 2 g)	8 (2.1)

30			Azithromycin (1 x 500 mg) + Cefoperazone (2 x 2 g)+ Ceftazidime	2 (0.5)
31			Azithromycin (1 x 500 mg) + Cefoperazone (2 x 2 g) + Ceftriaxone (1 x 2 g) + Meropenem (3 x 1 g)	2 (0.5)
32			Azithromycin (1 x 500 mg) + Cefoperazone (2 x 2 g)+ Meropenem (3 x 1 g)	6 (1.5)
33			Azithromycin (1 x 500 mg) + Cefoperazone (2 x 2 g) + Meropenem (3 x 1 g) + Ceftazidime (3 x 1 g)	2 (0.5)
34			Azithromycin (1 x 500 mg) + Levofloxacin (1 x 750 mg)+ Cefoperazone (2 x 2 g)	4 (1.1)
35			Azithromycin (1 x 500 mg) + Levofloxacin (1 x 750 mg) + Cefoperazone (2 x 2 g) + Cefotaxime (3 x 1 g)	1 (0.3)
36			Azithromycin (1 x 500 mg) + Levofloxacine (1 x 750 mg) + Cefoperazone (2 x 2 g) + Ceftriaxone (1 x 2 g)	6 (1.6)
37			Azithromycin (1 x 500 mg) + Levofloxacin (1 x 750 mg)+ Cefoperazone (2 x 2 g) + Meropenem (3 x 1 g)	32 (8.5)
38			Meropenem (3 x 1 g) + Ceftazidime (3 x 1 g)	1 (0.3)

39			Azithromycine (1 x 500 mg) + Levofloxacine (1 x 750 mg)+ Ceftazidime (3 x 1 g)	1 (0.3)
40			Azithromycine (1 x 500 mg) + Levofloxacine (1 x 750 mg) + Cefoperazone (2 x 2 g) + Meropenem (3 x 1 g) + Ceftazidime (3 x 1 g)	1 (0.3)
41			Azithromycine (1 x 500 mg) + Levofloxacine (1 x 750 mg)+ Cefoperazone (2 x 2 g) + Meropenem (3 x 1 g) + Ciprofloxacine (2 x 500 mg)	1 (0.3)
42			Levofloxacine (1 x 750 mg)+ Cefixime (2 x 200 mg) + Cefotaxime (3 x 1 g)	1 (0.3)
43			Levofloxacine (1 x 750 mg) + Cefoperazone (2 x 2 g) + Ceftriaxone (1 x 2 g)	1 (0.3)
44			Levofloxacine (1 x 750 mg) + Cefotaxime (3 x 1 g)	1 (0.3)
45			Levofloxacine (1 x 750 mg) + Ceftazidime (3 x 1 g)	1 (0.3)
46			Levofloxacine (1 x 750 mg) + Ceftriaxone (1 x 2 g) + Ceftazidime (3 x 1 g)	1 (0.3)
47			Levofloxacine (1 x 750 mg) + Meropenem (3 x 1 g) + Ceftazidime (3 x 1 g)	1 (0.3)
48			Levofloxacine (1 x 750 mg) + Meropenem (3 x 1 g) + Ciprofloxacine (2 x 500 mg)	1 (0.3)
49			Total	378
